# Modeling the Impact of White-Plague Coral Disease in Climate Change Scenarios

**DOI:** 10.1371/journal.pcbi.1004151

**Published:** 2015-06-18

**Authors:** Assaf Zvuloni, Yael Artzy-Randrup, Guy Katriel, Yossi Loya, Lewi Stone

**Affiliations:** 1 Israel Nature and Parks Authority, Eilat, Israel; 2 Department of Zoology, Tel Aviv University, Ramat Aviv, Tel Aviv, Israel; 3 The H. Steinitz Marine Biology Laboratory, Eilat, Israel; 4 Institute for Biodiversity and Ecosystem Dynamics, University of Amsterdam, Amsterdam, The Netherlands; 5 Department of Mathematics, ORT Braude College, Karmiel, Israel; 6 School of Mathematical and Geospatial Sciences, RMIT University, Melbourne, Australia; University of Chicago, UNITED STATES

## Abstract

Coral reefs are in global decline, with coral diseases increasing both in prevalence and in space, a situation that is expected only to worsen as future thermal stressors increase. Through intense surveillance, we have collected a unique and highly resolved dataset from the coral reef of Eilat (Israel, Red Sea), that documents the spatiotemporal dynamics of a White Plague Disease (WPD) outbreak over the course of a full season. Based on modern statistical methodologies, we develop a novel spatial epidemiological model that uses a maximum-likelihood procedure to fit the data and assess the transmission pattern of WPD. We link the model to sea surface temperature (SST) and test the possible effect of increasing temperatures on disease dynamics. Our results reveal that the likelihood of a susceptible coral to become infected is governed both by SST and by its spatial location relative to nearby infected corals. The model shows that the magnitude of WPD epidemics strongly depends on demographic circumstances; under one extreme, when recruitment is free-space regulated and coral density remains relatively constant, even an increase of only 0.5°C in SST can cause epidemics to double in magnitude. In reality, however, the spatial nature of transmission can effectively protect the community, restricting the magnitude of annual epidemics. This is because the probability of susceptible corals to become infected is negatively associated with coral density. Based on our findings, we expect that infectious diseases having a significant spatial component, such as Red-Sea WPD, will never lead to a complete destruction of the coral community under increased thermal stress. However, this also implies that signs of recovery of local coral communities may be misleading; indicative more of spatial dynamics than true rehabilitation of these communities. In contrast to earlier generic models, our approach captures dynamics of WPD both in space and time, accounting for the highly seasonal nature of annual WPD outbreaks.

## Author Summary

Coral reefs are deteriorating at alarming rates, with coral disease outbreaks increasing in prevalence and in space. Anomalously high ocean temperatures are thought to significantly contribute to this problem. We collected a unique and highly resolved dataset of a White Plague Disease (WPD) outbreak from the coral reef of Eilat (Israel, Red Sea). By fitting a novel epidemiological model to the data, we characterize the dynamics of WPD, and study the possible effects of future increasing sea-surface temperatures (SST) on disease dynamics. In contrast to earlier studies, our approach captures the dynamics of coral disease both in space and time, and accounts for the highly seasonal nature of the annual outbreaks. We also apply a novel combination of spatiotemporal statistics and null hypothesis approaches to study the disease progression. Model forecasts into the future show that for some scenarios even an increase of only 0.5°C in SST can cause epidemics to double in magnitude. Since the probability of infection is found to be negatively associated with coral density, this implies that the spatial nature of disease transmission can both enhance and restrict the magnitude of annual epidemics. The results have implications for designing management policies appropriate for coral reef conservation.

## Introduction

Infectious diseases are recognized as important factors affecting community structure and dynamics in scleractinian corals [1]. They can result in a significant reduction in live coral coverage [2] and density [3], and in extreme cases are able to initiate coral-algal phase-shifts through mortality of key reef-building corals and consequent changes to the reef framework [2, 4]. In the Caribbean, disease outbreaks are considered to be one of the primary causes of the accelerating destruction of the reefs [5–9].

Patterns of coral diseases in space and time are related to various environmental parameters [10]. Several studies have shown that disease prevalence and transmission rates are significantly associated with high water temperatures [11–16], high UV radiation [17], decline in water quality [18–22], vector and host densities [13, 23–26], and intensity of coral bleaching [13, 27–32]. However, the relative contributions of various environmental factors to coral disease dynamics are likely to be complicated and synergistic [33–35].

We are living in an era of rapidly changing climate [34, 36], where anomalously high temperatures are becoming a significant environmental factor affecting the health and resilience of coral reefs [37]. Some coral diseases display inter-annual fluctuations, where intense epidemics are coincident with periods of anomalously high seawater temperatures [13, 36, 38–41]. In addition, several coral diseases, such as black band (e.g., [15, 16, 22, 42–44]), white plague (e.g., [45]), white syndrome [13, 32], ulcerative white spots [46], *aspergillosis* [47] and white pox [48], display clear seasonal variations in their intensity, with higher prevalence, severity or progression during summer months.

Mathematical and statistical modeling are important tools for understanding coral disease dynamics [35, 49], and in light of global change, there is obvious interest in assessing long-term effects that variations in seawater temperatures will have on the intensity of coral diseases, as well as their impact on the reef. However, the study of disease dynamics in natural populations is often difficult and in many cases diseases are likely to display complex interactions between extrinsic forcing, due to environmental effects (such as elevated temperatures), and intrinsic dynamics, due to the interplay of epidemiological variables (such as susceptibility and patterns of transmission in space and time; [34]). Therefore, when attempting to understand disease dynamics, it is important to take into account both epidemiological and environmental variables.

**Fig 1:**
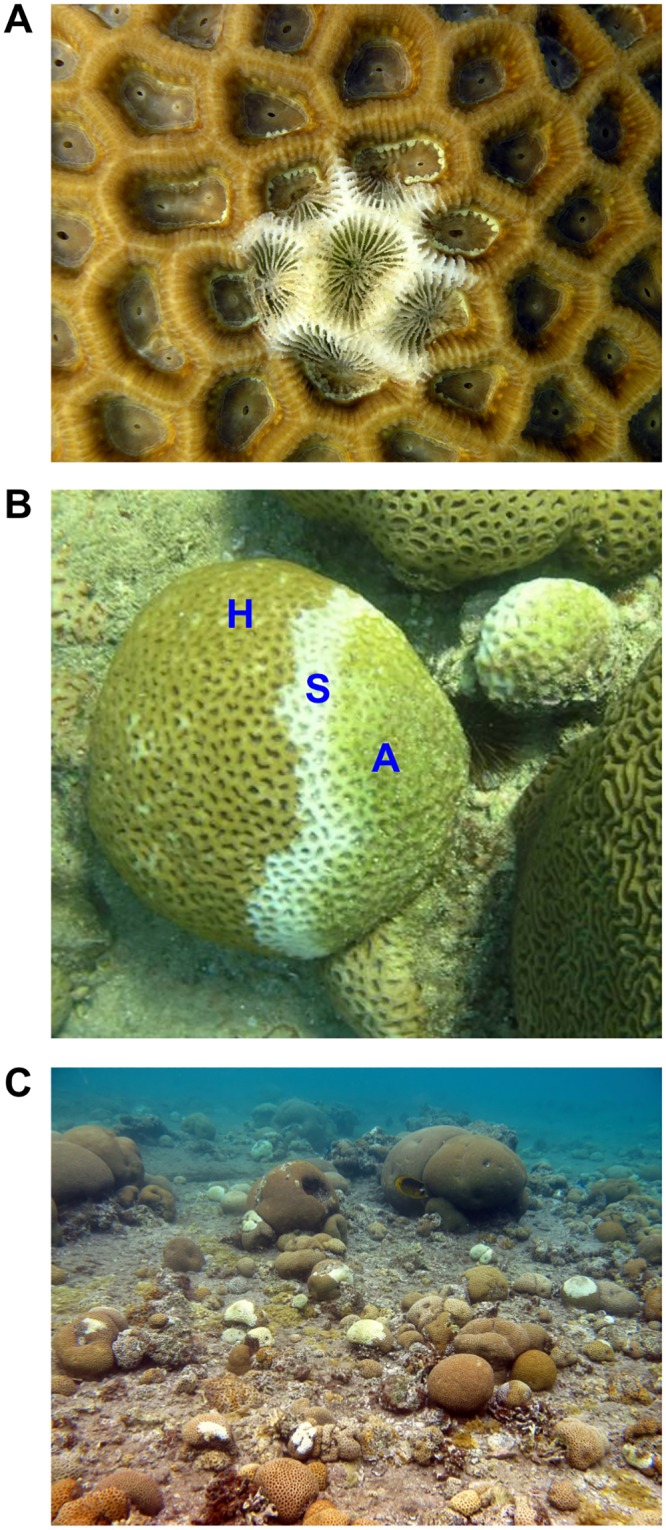
Coral colonies from the genus Favia infected with WPD (white-plague disease). **A**) Initiation of WPD—a thin zone of bleached tissue grading into exposed coral skeleton. **B**) A sharp boundary between apparently healthy tissue (‘H’) and freshly exposed skeleton (‘S’), with no build-up of microorganisms or necrotic tissue visible to the eye. With time, the exposed skeleton becomes colonized by algae (‘A’). **C**) An aggregation of corals infected with WPD.

In this study we focus on white-plague disease (WPD; see Fig 1) in the Red Sea. Throughout the Caribbean and Western Atlantic, WPD is recognized as a destructive coral infectious bacterial disease [50, 51] that affects a number of reef framework-building coral species [3, 52–54]. Infected corals exhibit a rapid rate of tissue degradation of up to two centimeters per day and the existence of the disease on the reef results in a clumped distribution of infected individuals [50]. The Gram-negative bacterium *Aurantimonas coralicida* gen. nov. sp. nov. was identified as the causative agent of WPD in the Caribbean [55].

WPD has become commonly observed in the Red Sea since at least 2002 [52]. A sharp line between apparently healthy tissue and a thin zone of bleached tissue grading into exposed coral skeleton are the typical signs of the disease in this region (see Fig 1), similar to the signs observed in the Caribbean [50]. In contrast to the Caribbean, however, the causative agent in the Red Sea is known to be *Thalassomonas loyana* sp. Nov. [56]. Aquarium experiments have shown that WPD in the Red Sea is an infectious disease [52, 57]. However, the transmission pattern of the disease in natural coral communities has not been quantitatively studied, and to date there is no data regarding the local transmission mode of this disease within coral communities. In addition, we do not know how the transmission strength of WPD varies in accordance to the season and what will be the future impact of the disease on coral communities under different climate change scenarios.

In order to address these questions, we collected a unique dataset that characterizes the dynamics of WPD in space and time within a large pool of corals at the Eilat coral reef (Israel, Red Sea). The study extended between June 2006 until May 2007 with monthly samples that provided twelve spatial “snapshots” of the reef. The principal idea behind much of our modelling rests on analyzing the evolving relationship between the number of Newly Infected Corals (NICs) found in a particular month to the number of Previously-Infected corals (PICs) found in the month before. A novel epidemiological model is developed, taking into account spatial, temporal and environmental parameters, which we find play a role in the transmission of WPD. The model is used to determine those factors which control the transmission of WPD through the coral population. In contrast to other recent efforts to model coral diseases (e.g., [49, 58]), our model captures the dynamics of WPD in space and time, while also taking into account climate drivers and the highly seasonal nature of annual WPD outbreaks. The model characterizes the dynamics of the epidemic as it occurs in time. These features of the model prove to be of the greatest significance when predicting the future impact of coral diseases on local coral communities. Other modelling studies that we are aware of [e.g., 49,58] attempt to model the annual average numbers of infected corals, which is the coarsest of descriptions possible when attempting to characterize an epidemic.

### Study Site

The study site was located at the shallow water reef (depth of *ca*. 1.5 m) off the shore of the Interuniversity Institute (IUI) in Eilat. The reef is relatively uniform with respect to bathymetry and is situated on a gentle slope (*ca*. 3°) on flat beach-rock. The reef did not appear to exhibit any particular dominant water-flow direction due to the high impact of the erratic and changing wave action. The reef is characterized by relatively high coral density, which allows for a relatively large number of infection cases per unit area. Thus the IUI site is particularly suitable for studying the spatial distribution and the dynamics of coral diseases.

### Field Sampling

A 10×10 m plot was surveyed once a month, from June 2006 until May 2007 providing twelve “snapshots” in total. The size of the plot and the period of time between snapshots were based on a preliminary survey where we roughly assessed the clustering size of infected corals, and the development time of new infections. The four corners of the plot were marked in the field, and a grid made of ropes and elastic bands was placed on the plot dividing the plot to 100 subunits of 1×1 m. Using photography (photoquadrats), all 2,747 susceptible corals within this area were mapped and an X-Y coordinate of the coral’s centre within the plot was allocated, following the “*center rules*” of Zvuloni *et al*. [59]. Once a month, the grid was placed precisely on the same area and the locations of infected corals were recorded. Corals were classified in the field as infected if they showed typical signs of the disease—a sharp line between apparently healthy tissue and a thin zone of bleached tissue grading into exposed coral skeleton (Fig 1, [50]) and some level of progression (i.e., increased severity) relative to the previous snapshot. The Israel National Monitoring Program of the Gulf of Eilat provided continuous measurements of sea-surface temperature (SST), *ca*. 20 m away from the plot, as obtained from two temperature probes (Campbell Scientific, Temperature Probe Model 108; accuracy of ±0.1°C within the range of 20–30°C and resolution of 0.1°C).

### Spatiotemporal Patterns of WPD

The 12 spatial snapshots of the reef-section were organized as eleven pairs of sequential snapshots, where in each pair infected corals were partitioned into two groups:

1. Newly-Infected Corals (NICs)—those corals that had signs of infection in the current snapshot, but not in the previous one.
2. Previously-Infected Corals (PICs)—those corals that were infected in the previous snapshot.

Our conjecture was that if inter-colonial transmission is significant for the spread of the disease, NICs should develop in closer proximity to PICs than would be expected at random. To test this hypothesis, we developed a simple, but novel, spatiotemporal index, which is based on Ripley’s *K*-function [60, 61]. While the *K*-function tests the spatial pattern of a single group of events, our spatiotemporal index *n*(*r*) was designed to test the spatial relations between two groups of events, in this case two groups of infected corals—the NICs and the PICs. This index is defined as the mean number of NICs in a given month within a radius *r* from a PIC of the previous month, and is calculated as:

$$n\left(r\right)=\frac{1}{m}\sum_{i=1}^{m}\sum_{j=1}^{k}I_{r}\left(d_{ij}\right).$$ (1)

Here, *m* and *k* are the numbers of PICs and NICs, respectively, in the tested pair of sequential sampling dates, *d*_*ij*_ is the distance between any PIC *i* and NIC *j*. The indicator variable *I*_*r*_(*d*_*ij*_) indicates whether or not NIC *j* is located within radius *r* from PIC *i*. Thus, *I*_*r*_(*d*_*ij*_) receives a value of 1 if *d*_*ij*_ < *r* and zero otherwise. In contrast to the nearest-neighbor approach used by Zvuloni *et al*. [16] to identify whether NICs form aggregations in the vicinity of PICs, the *n*(*r*) index also quantifies the spatial scale of aggregation, as it is calculated for a range of distances *r* (similarly to Ripley’s *K* function; see Ripley [60, 61]). Using a null model approach, which bases the null expectation on the spatial distribution of the entire pool of susceptible corals, we ascertained whether the *k* NICs found in the field were significantly aggregated around the PICs (see Material and Methods).

### Spatiotemporal Epidemic Model

We model disease transmission by using a stochastic spatiotemporal model similar to Zvuloni *et al*. [16], but with a new maximum-likelihood fitting procedure to estimate model parameters from the field-data. The analysis that follows is based on the classical Susceptible-Infected-Susceptible (SIS) model of epidemiology [62, 63]. Corals are classified as either susceptible or infected. A susceptible coral can become infected when the disease is transmitted from a (usually) neighboring PIC, and an infected coral can return to be susceptible if the disease stops showing clinical signs. The model assumes transmission is via local waterborne infections (i.e., susceptible corals are infected by suspended infectious material originating from diseased corals within the study site). The assumptions underlying the construction of the model are that: (i) there is a higher probability that infection events take place in close proximity to existing infections; and (ii) there is a cumulative impact of multiple infections on a single susceptible coral, such that the more infected neighbors a susceptible coral has, the more likely it is to become infected itself.

More specifically, the model determines the probability of each susceptible coral being infected and thus becoming a Newly Infected Coral (NIC). The probability of being infected by any Previously Infected Coral (PIC) within the study site is assumed to be inversely proportional to the distance (*d*) of the PIC. In addition, a susceptible coral can be infected by any of the PICs present. Thus, we define the probability of a coral *i* (from all susceptible corals within the study site) to become infected during a month *t* (1≤*t*≤11; in total there are eleven sequential sampling dates) as:

$$p_{t}\left(i\right)=c_{t}\overset{}{\underset{j\in PIC_{t}}{\sum }}\frac{1}{d_{ij}^{\alpha }},$$ (2)

where *PIC*_*t*_ is the set of all PICs in month *t* and *d*_*ij*_ is the Euclidean distance between coral-*i* and PIC-*j*. The exponent *α* characterizes the decay of the transmission probability with distance. In this way, infections are preferentially passed to neighboring susceptible corals. Another special feature of the model is the inclusion of seasonal drivers [64] through the constants *c*_*t*_ that characterize the transmission strength of WPD in each month *t*. These constants presumably depend on environmental factors that change in accordance to the season (e.g., seawater temperatures), and therefore may link between the spatiotemporal model and these factors. Note that all PICs within the study site influence the probability of any susceptible coral to become infected. The definition ensures the probability is inversely proportional to the coral’s distance from any PIC. In addition, the probability increases with the number of PICs and the increase will be largest for neighboring PICs (where the distances *d*_*ij*_ are small).

### Estimating the Best Fitting Parameters

Model parameters that need to be estimated are: (i) the exponent *α* that characterizes the decay of the transmission probability with distance, and (ii) the constants *c*_*t*_ that characterize the transmission strength in each month *t*. In order to find the best fitting parameters *α*, *c*_1_, …, *c*_11_, we define a likelihood function and then maximize it with respect to these parameters.

Given *PIC*_*t*_ (the set of PICs in month *t*), the probability that the set of corals infected during this month is precisely the set *NIC*_*t*_ of NICs is:

$$p\left(NIC_{t}|PIC_{t},\alpha ,c_{t}\right)=\prod_{i\in NIC_{t}}p_{t}\left(i\right)\times \prod_{i\notin NIC_{t}\cup PIC_{t}}\left(1-p_{t}\left(i\right)\right).$$ (3)

Here, the first term on the RHS is the probability that all the corals in the set *NIC*_*t*_ are infected, and the second product is the probability that all the corals, which are *neither* in the set *NIC*_*t*_, nor in the set *PIC*_*t*_, are *not* infected.

The total probability of obtaining the empirical results given the model, that is the likelihood function, is thus given by:

$$L\left(\alpha ,c_{1},\ldots ,c_{11}\right)=\prod_{t=1}^{11}\left[\prod_{i\in NIC_{t}}p_{t}\left(i\right)\times \prod_{i\notin NIC_{t}\cup PIC_{t}}\left(1-p_{t}\left(i\right)\right)\right],$$ (4)

and the log-likelihood is given by:

$$LL\left(\alpha ,c_{1},\ldots ,c_{11}\right)=\overset{11}{\underset{t=1}{\sum }}\left[\underset{i\in NIC_{t}}{\sum }\text{log}\left(c_{t}\underset{j\in PIC_{t}}{\sum }\frac{1}{d_{ij}^{\alpha }}\right)+\underset{i\notin NIC_{t}\cup PIC_{t}}{\sum }\text{log}\left(1-c_{t}\underset{j\in PIC_{t}}{\sum }\frac{1}{d_{ij}^{\alpha }}\right)\right]$$ (5)

The maximum-likelihood estimate for the parameters is obtained by maximizing the function in Eq 5. The procedure described below reduces the multi-variable optimization problem to a series of one-dimensional problems. We note that since each of the variables *c*_*t*_ appears in only one of the summands, we find that:

$${\text{max}}_{\alpha ,c_{1},\ldots ,c_{11}}LL\left(\alpha ,c_{1},\ldots ,c_{11}\right)={\text{max}}_{\alpha }M\left(\alpha \right)$$ (6)

where:

$$M\left(\alpha \right)=\sum_{t=1}^{11}{\text{max}}_{c_{t}}\left[\sum_{i\in NIC_{t}}\text{log}\left(c_{t}\sum_{j\in PIC_{t}}\frac{1}{d_{ij}^{\alpha }}\right)+\sum_{i\notin NIC_{t}\cup PIC_{t}}\text{log}\left(1-c_{t}\sum_{j\in PIC_{t}}\frac{1}{d_{ij}^{\alpha }}\right)\right].$$ (7)

The profile likelihood function *M*(*α*) is the maximum of *LL* with respect to *c*_1_, …, *c*_11_ with a fixed *α*. In order to maximize *LL*, we proceed as follows in our numerical algorithm:

(i) We step *α* incrementally through a certain interval in small steps. For each of the *α* values we run over *t* from 1 to 11 (the number of pairs of sequential sampling dates), and for each of the values of *t* we numerically find *c*_*t*_ = *c*_*t*_(*α*) that maximizes:

$${\tilde{M}}_{t}\left(\alpha ,c_{t}\right)=\sum_{i\in NIC_{t}}\text{log}\left(c_{t}\sum_{j\in PIC_{t}}\frac{1}{d_{ij}^{\alpha }}\right)+\sum_{i\notin NIC_{t}\cup PIC_{t}}\text{log}\left(1-c_{t}\sum_{j\in PIC_{t}}\frac{1}{d_{ij}^{\alpha }}\right).$$ (8)

(ii) We use these eleven values to obtain:

$$M(\alpha )=\sum_{t=1}^{11}{\tilde{M}}_{t}(\alpha ,c_{t}(\alpha )).$$ (9)

(iii) We then find the value $\hat{\alpha }$ for which *M*(*α*) is maximal. The maximum likelihood estimate for the parameters is then $\left(\hat{\alpha },\;c_{1}(\hat{\alpha }),\ldots ,c_{11}(\hat{\alpha })\right)$.

### Model Validation and Null Hypothesis Approach

Two approaches were used to test the null hypothesis that the observed data is generated by the SIS epidemic model driven by Eq 2:

1. The number of NICs observed in the field (*k*) in each month was compared to the distribution of the simulated number of NICs generated from 1,000 model realizations using the best-fitting parameters *α*, *c*_1_, …, *c*_11_.
2. The model fit was tested by comparing the spatiotemporal index *n*(*r*) (Eq 1) calculated for the actual data with that generated by repeated model realizations using Eq 2.

For further details see Material and Methods.

### Predicting the Future Impact of Coral Diseases

We link the model to seawater temperatures and test possible effect of increasing temperatures on disease dynamics. By controlling the temperature we can test different climate change scenarios. Our model differs from the usual mean-field SIS models in which susceptible individuals and infectives mix randomly and in a uniform manner; here an explicit spatial component is incorporated through the use of Eq 2. For all future projections, we use the last month of the real data as initial conditions. Then, at each monthly time step, the susceptible corals that become infected over the coral network are stochastically determined according to Eq 2, given the spatial compositions of the sampled community. The computations keep track of which of all the corals become infected and which remain susceptible. Two different demographic assumptions were applied in the simulations—(i) constant influx of recruits, and (ii) free-space regulation of recruitment (see Material and Methods).

## Results

In total, 2,747 susceptible corals were observed and mapped within the surveyed 10×10 m plot. 85% of the corals belonged to the genus *Favia*, 14.3% *Platygyra*, 0.6% *Favites* and 0.1% *Goniastrea*. The coral community is extremely dense (>50 corals/m^2^) and mostly composed of relatively small massive corals (see S1 Fig), many of which are susceptible to infection by WPD. The cumulative number of corals infected with WPD within the studied year, from June 2006 until May 2007, was 120 (*ca*. 4.4% of the susceptible corals). Among these corals, 64 (53.3%) died, 44 (36.7%) survived (i.e., some level of partial mortality was caused, but the disease stopped showing any clinical signs and progression) and 12 (10%) remain infected until the end of the survey. Bleached colonies were not observed on this reef and Black Band Disease (BBD) was observed at relatively low prevalence [< 0.8%; see Zvuloni *et al*. [16]].

### Spatiotemporal Pattern of WPD

**Fig 2:**
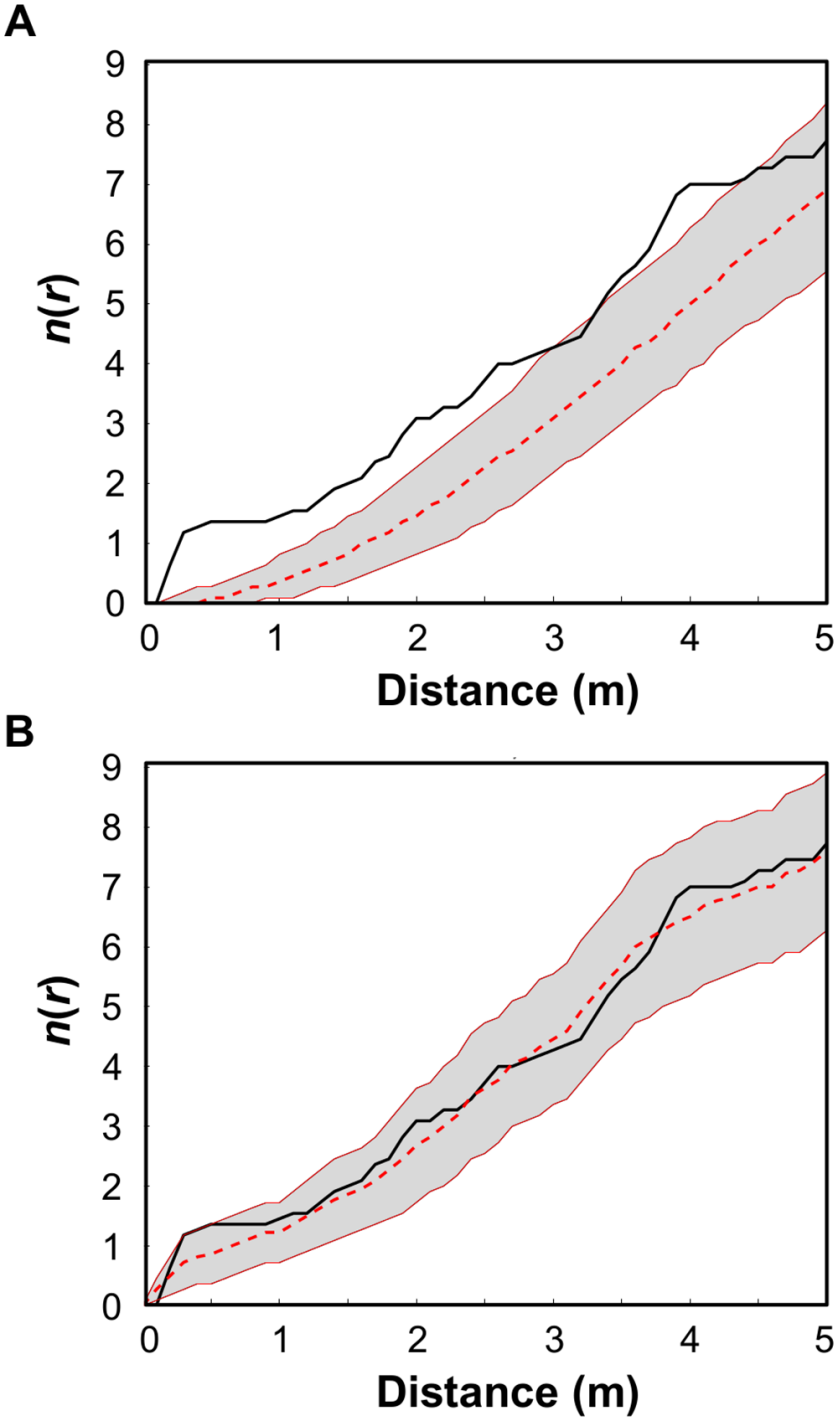
Plots of the spatiotemporal index *n*(*r*), calculated for pairs of sequential sampling dates (here June-July; see text). The black line represents the observed *n*(*r*) values (Eq 1) for corals infected with WPD (white-plague disease), the solid red lines bound the Monte Carlo 95% CI envelope for two different null expectations, and the dashed red line marks the median of these: **A)** new infections develop randomly within the studied plot, independent of the spatial location of infected corals from the previous month; and **B)** new infections develop according to the spatiotemporal model (Eq 2). For distance scales *r* where *n*(*r*) values fall within the envelope, the spatial distribution of infected corals does not differ significantly from the null expectation. Infected corals are significantly more aggregated where the observed *n*(*r*) values fall above the CI envelope. Comparisons between all the other pairs of sequential sampling dates are given in S2 Fig.

Based on our analysis with the sptiotemporal index n(r) (eq 1), we found that in all cases Newly-Infected Corals (NICs) appeared to form aggregations around Previously-Infected Corals (PICs) over distance scales of up to 4.5 m (see e.g., Fig 2A for June-July 2006, and S2a Fig for all eleven sequential snapshots). This is because the index n(r) of the observed data sits almost always above the Monte Carlo 95% CI envelope generated by the null test (see Materials and Methods). That is, in all cases the hypothesis that the NICs were infected by a random process of disease transmission, independent of the spatial location of the PICs, was rejected. In S3 Fig we provide spatial illustrations of the disease dynamics over the studied year showing the spatial relation between PICs and NICs.

### The Best-Fitting Model Parameters

**Fig 3:**
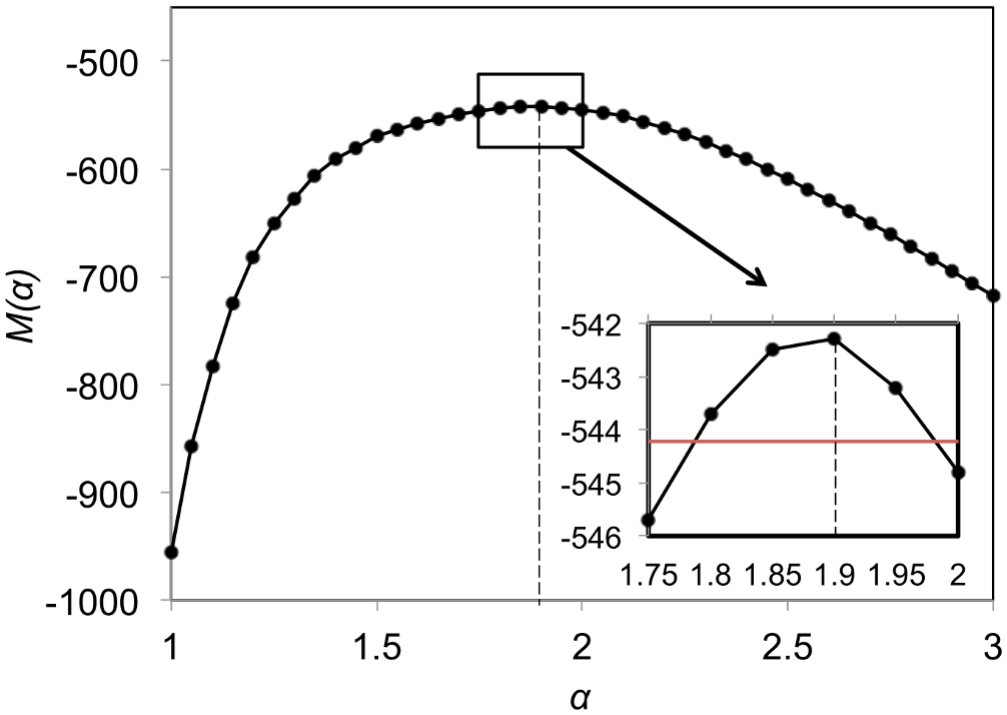
Profile likelihood function *M*(*α*). The function is maximized at $\hat{\alpha }$ = 1.9, giving the estimate of parameter *α*. The insert shows a close up of the 95% CI of *α* (represented by the red horizontal line).

Using the maximum likelihood fitting procedure, the best-fitting exponent *α*, which in Eq 2 expresses the decay of the transmission probability with distance, was found to be $\hat{\alpha }$ = 1.9 (Fig 3). The maximum-likelihood estimates for the best-fitting parameters *c*_*t*_, constants that express the transmission strength of the disease during month *t* (*c*_1_, …, *c*_11_) and presumably depend on environmental factors, are given in S1 Table.

### Validity of the Spatiotemporal Model

**Fig 4:**
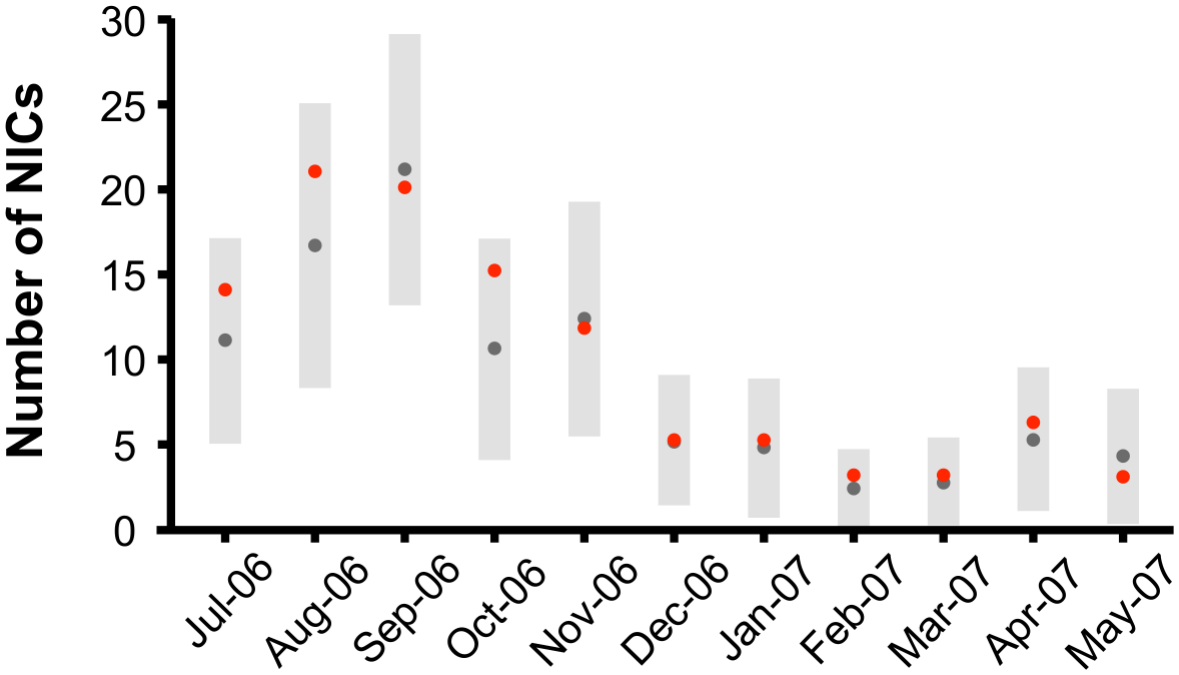
Number of newly-infected corals (NICs). The red dots represent the number of NICs observed in the field along the studied year. The grey dots represent the median number of NICs as predicted by generating infections according to the SIS epidemic model based on Eq 2 (see text), and the grey bars represent their 95% confidence interval.

For all pairs of sequential sampling dates, the number of NICs observed in the field (*k*) was within the 95% confidence interval (CI) envelope of the simulated number of NICs obtained from the model realizations (Fig 4). We thus could not reject the hypothesis that the observed NICs were produced according to Eq 2. (Note that here we are essentially testing the model’s “goodness of fit” to the data, and thus there is no need to use the first half of the time series to predict the second half.)

Additional support for the validity of the spatiotemporal model is that in nearly all cases the observed *n*(*r*) was purely within the null expectation of the model for all distance scales *r* (e.g., Fig 2B). However, in a few cases the observed *n*(*r*) was found to be greater than the upper bound of the 95% CI envelope generated by the model realizations for certain distance scales (see, for example, August-September 2006 in S2b Fig).

### Seasonal Patterns and the Epidemic Potential of WPD

**Fig 5:**
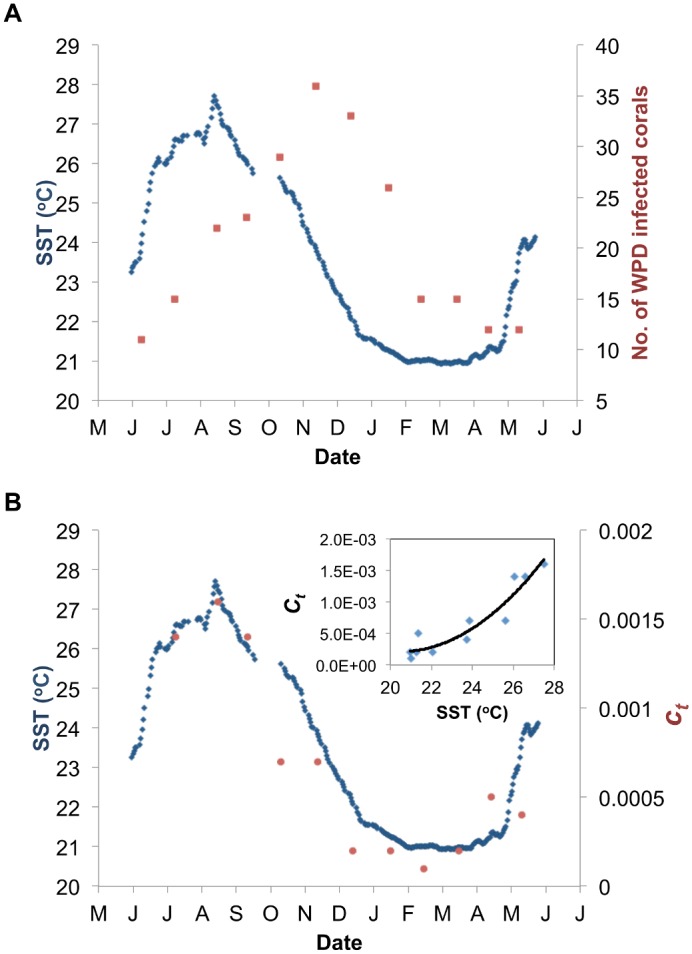
Seasonal pattern of WPD. **A)** Number of corals infected with white-plague disease (WPD) within the studied plot (red squares), and **B)** the estimated parameters **ct** (red circles) which express the transmission strength of the disease (see Eq 2), as opposed to sea-surface temperature (SST; 7 days running average; blue line) starting from June 2006 to May 2007. Polynomial regression between *ct* and SST is shown in the insert.

The number of infected corals observed within the study site ranged from a low of 11 infected corals during June 2006 to a peak of up to 36 infected corals in November 2006 (Fig 5A). The disease prevalence lagged *ca*. 3 months behind the sea surface temperature (SST) that reached its seasonal peak of 27.7°C at the end of August 2006. On the other hand, we found a high association between SST and *c*_*t*_ (see Fig 5B; Adjusted *r* ^2^ = 0.88, goodness of fit is SSE: 3.02e-07, RMSE: 0.0001943), which is expressed by the polynomial relationship:

$$c_{t}=p_{1}\cdot SST^{2}+p_{2}\cdot SST+p_{3}$$ (10)

having coefficients [with 95% CI]: *p*_*1*_ = 2.968e-05 [-6.95e-06, 6.631e-05], *p*_*2*_ = -0.001216 [-0.002972, 0.0005402] and *p*_*3*_ = 0.01267 [-0.008197, 0.03353].

We calculated the epidemiological reproductive number *R*_0_ [65] for the time period between June and August 2006, when the cumulative incidence of infections grows approximately exponentially with time (see Material and Methods). The result shows that the development of the disease within the coral community resulted in an epidemic-like growth with *R*_0_ = 1.2 (*r* = 0.35; *T*_*G*_ = 0.53).

### Long-Term Impact of WPD and Implications of Climate Change

**Fig 6:**
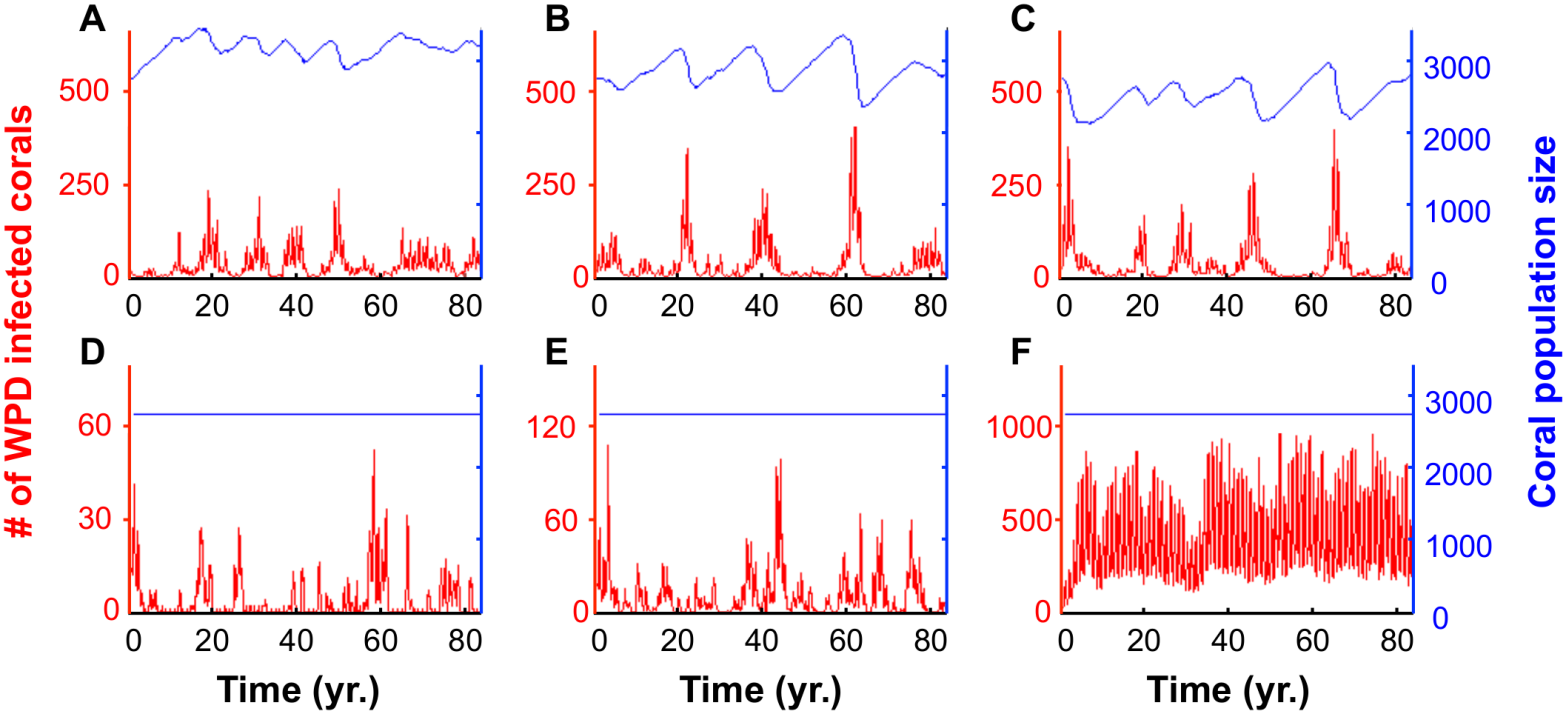
Simulated future projections of the local coral community spanning 80 years. The future projections in panels **A**, **B** and **C** rely on the demographic scenario of constant influx of recruits (64 recruits per year). Panels **D**, **E** and **F** rely on the scenario of free-space regulation of recruitment (see Material and Methods). Panels **A** and **D** are based on the SST time-series measured between June 2006 and May 2007 recurrently from year to year in the corresponding months. Based on this time-series, we generate future projections by adding 0.5°C (panels **B** and **E**) and 1°C (panels **C** and **F**) to the SST of each month. In these simulations we allow each new recruit to settle randomly anywhere on the 10×10 m plane. S4 Fig demonstrates robustness of these patterns under mild parameter variations.

The unexpected high association found between SST and the transmission strength *c*_*t*_ of WPD (Adj. *r* ^*2*^ = 0.88; see Fig 5B) extracted from fitting the spatiotemporal model to the data allows us to assess the potential long term impact of WPD on the local coral community under different climate change scenarios. We first examine model projections assuming that there is no climate change and that the seasonal cycle of SST temperature repeats in exactly the same way from year to year. Projections of the disease 80 years into the future under these conditions (see Material and Methods) show seasonally driven annual cycles (Fig 6A and 6D). Indeed, each year the transmission strength of the disease increases as SST rises from March to August, and then rapidly decreases from September to February (Fig 5B).

We then considered the impact of a general mean increase in SST assuming a scenario of constant influx of recruits (“recruitment limited”). Fig 6B shows the effects of increasing SST by 0.5°C while Fig 6C shows the effect of only a 1°C increase. We find multi-annual cycles, in which severe epidemics take place every few years when the density of susceptibles corals build up to relatively high levels (Fig 6A, 6B and 6C). The intensity of these epidemics increases with increasing SST, but their frequency is still restricted by the rate at which corals are replenished.

The same simulations were examined under an assumption that the coral community is governed by space limitation and is thus “free-space regulated,” or dependent on the level of free substrate available in the local patch. This follows from the hypothesis that space is a limiting resource in many marine benthic populations [66–69]. Fig 6D, 6E and 6F show that under a scenario of free-space regulation of recruitment, a mean increase of only 0.5°C can cause epidemics to double in size, while a mean rise of 1°C can cause increases scaling in orders of magnitude.

Finally, we point out that these model “forecasts” should not be viewed as accurate predictions of monthly changes but more as qualitative guidelines as to what might be expected should there be a future long-term trend in SST temperatures. This corresponds to the “strategic” approach suggested by May [70], which “sacrifices precision in an effort to grasp at general principles. Such general models, even though they do not correspond in detail… provide a conceptual framework for discussion and further exploration”.

## Discussion

Our work offers the very first model fit of any coral disease epidemic, over the timescale of the epidemic, to be found in the literature. Other attempts failed to succeed either because they did not have the fine resolution data (e.g., 12 monthly sampling points) over the timespan of the epidemic, and/or because they did not have a modelling formulations to conduct parameter estimates and model fits. At best other modelling attempts have only taken into account the total annual numbers of infected corals, which is the coarsest of descriptors when characterizing epidemic dynamics.

In the beginning of the transmission season, the spread of the disease in the local community exhibited epidemic-like growth motivating us to study *R*_0,_ the epidemiological reproductive number. *R*_0_ was estimated (see Material and Methods) for the time period between June and August 2006 (the development period of the disease within the community) and was found to be greater than unity (*R*_0_ = 1.2; *r* = 0.35, *T*_*G*_ = 0.53). This value of *R*_0_ was lower than these calculated for BBD for the outbreaks of 2006 and 2007 (*R*_0_ = 1.6 and 1.7, respectively; [16]). In BBD, both the exponential growth rate (*r*) and the mean generation interval of the epidemic (*T*_*G*_) were greater than these calculated for WPD. Although the observed seasonal outbreak generated an epidemic-like growth, the disease did not spread over a large fraction of the susceptible corals (see Fig 5). Our model simulations suggest that seasonality and low *R*_0_ are not the only factors responsible for this restriction in disease spread, and in particular, that the spatial component of the system may also play a significant role.

The spatial scale of aggregations of NICs in the vicinity of existing infected corals indicates that small-scale inter-colonial transmission is significant within the community under study (see Figs 2 and S2). That is, infected corals are ‘hotspots’ of potentially infectious material, being transmitted to nearby susceptible corals on the reef (see S3 Fig). We find that the larger the number of infected corals in proximity to a susceptible coral, and the closer they are, the higher the likelihood of this coral becoming infected itself. Similar results were found in previous studies for WPD in the Florida Keys [50], for BBD in the Red Sea [16] and for *aspergillosis* in the Caribbean [71].

These findings are in contrast to a recent study by Muller & van Woesik [72] which suggests that coral diseases in the Caribbean do not follow a contagious-disease model. One possible explanation for the inconsistency in the results between these studies, is that there are differences in the infection process of the two identified pathogens (i.e., the causative agents are known to be different between regions). In addition, coral communities across the Red Sea are much denser than in the Caribbean; while in the present study 2,747 corals susceptible to WPD were recorded within a 10×10 plot, Muller & van Woesik [72] recorded only 78±12 (mean±SE) susceptible corals within the same plot size. Hence, the average distance among susceptible corals in the Caribbean is much greater than in Eilat, making the probability of identifying inter-colonial transmission significantly lower than in Eilat. As such, the findings of Muller & van Woesik [72] would not necessary contradict the findings of our transmission model. In a similar spirit, Bruno *et al*. [13] also argue that high coral cover and/or density increases the occurrence for horizontal transmission of White Syndrome between corals across the Great Barrier Reef in Australia.

Testing the goodness of fit of our spatiotemporal model (Eq 2) in two different ways [e.g., distribution of NICs and clustering index *n*(*r*)] reveals that in all cases the model could effectively predict the number of NICs and in nearly all cases it could simulate the actual spatial patterns of new infections. However, in a few cases the observed *n*(*r*) was found to be greater than the upper bound of the 95% CI envelope generated by the model realizations for certain distance scales. These deviations suggest that there may be mechanisms involved in the transmission process that are not fully captured by our simple model. However, by comparing the results obtained from the random simulated infections (S2a Fig) and those obtained from the spatiotemporal model (alongside with S2b Fig), it is clear that the spatiotemporal model always outperformed the random transmission model.

The unexpectedly high association found between SST and the transmission strength *c*_*t*_ of WPD (Adj. *r* ^*2*^ = 0.88; see Fig 5B) extracted from fitting the model to the data indicates the power of the modeling approach. This association strongly suggests that SST is the seasonal driver behind the WPD dynamics, and might well be explained by the response of the host and/or pathogen to seasonal thermal fluctuations. High seawater temperatures may cause stress to coral hosts and increase their susceptibility to disease infections [73], while at the same time they may increase the virulence of the pathogen [74]. Previous studies from other locations have also identified clear seasonal patterns of various coral diseases, such as white syndrome [13, 32], BBD [15, 22], ulcerative white spots [46], *aspergillosis* [47] and white pox [48], related particularly to warm seawater temperatures. In this study, the seasonal patterns of the transmission strength of WPD (*c*_*t*_) preceded the seasonal patterns of the disease prevalence by *ca*. three months (see Fig 5B vs. Fig 5A, respectively). This suggests that the high seawater temperatures may directly affect the susceptibility of the corals and/or the virulence of the pathogen, but indirectly affect the prevalence of WPD. That is, the impact of the disease on the reef might be the lagged response (*ca*. three months) to processes that advance the progression of the disease within and among coral colonies.

The strong coupling of the transmission strength of the disease (measured by *c*_*t*_) and the seasonal variation in SST, forms the basis for our forecasts of future global warming scenarios. The association suggests that the higher seawater temperatures associated with future global warming will intensify the impacts of WPD on reefs. Our future predictions verify that in a demographic scenario, when recruitment is purely free-space regulated, such that the coral community density is relatively constant in steady-state conditions, a mean increase of only 0.5°C can cause epidemics to double in size. Likewise, a mean rise of 1°C can even lead to increases in several orders of magnitude. However, in reality, the influx of recruits is likely to be limited to some extent and located along a continuum between the two extremes (i.e., constant influx vs. free-space regulation). Thus, it is reasonable to assume that during an intense epidemic, when many susceptible corals will be removed through death, the spatial component of the disease will play a role in the disease dynamics.

Indeed, our future predictions confirm that the spatial component of the disease transmission system has, to some extent, a protective effect that restricts the magnitudes of annual epidemics. Under a demographic scenario of constant influx of recruits, the mean coral community densities decrease as the SST increase (Fig 6A, 6B and 6C). In this case the intensity of the disease does not change with increased SST scenarios. We suggest that this is because the decrease in density discounts for the increase in the transmission strength of the disease (i.e., each of these parameters work in a different direction). In practice, the decrease in coral density increases the mean distance between infected and susceptible corals within the community and thus decreases the potential for disease transmission [13]. Such a positive relationship between host density and disease transmission has been demonstrated in many host-pathogen systems [75–78], and is considered as an important property of the infectious process [79]. Specifically with infectious coral diseases, high coral density may have similar effects to that of high coral coverage; effectively this reduces the mean distance between neighboring corals, and as with our spatiotemporal epidemic model, increases the likeliness of inter-colonial transmission. Indeed, Bruno *et al*. [13] demonstrated that for white syndrome outbreaks to occur in the Great Barrier Reef in Australia, in addition to thermal stress, coral coverage must be relatively high (50% or higher).

Our model suggests that an infectious disease, such as WPD in the Red Sea, cannot lead to a complete destruction of the coral community, due to the spatial nature of the disease transmission and its protective effect. However, this also implies that signs of recovery of local coral communities may be misleading, and are not truly indicative of their rehabilitation (see for example the sharp fluctuations in the disease prevalence in Fig 6C). In addition, environmental changes, such as increasing levels of SST, can shift the nature of recruitment on local scales, altering the way in which the spatial component of the system restricts or enhances local disease dynamics. In addition, note that the remarkable transition in disease prevalence, which is observed when recruitment is free-space regulated (Fig 6D, 6E and 6F), may indicate that the interaction of the seasonal driving force and the spatial nature of the system has higher levels of complexity, beyond those described here. These more complex aspects of this system are beyond the scope of the present paper.

To summarize, we have addressed some fundamental questions regarding the dynamics of WPD in the Red Sea. Spatiotemporal statistics combined with null hypothesis approaches proved to be effective tools for understanding epizootiological processes in coral reef communities. The new spatiotemporal index, *n*(*r*), proved to be specifically tuned to detect the localized transmission dynamics among the infected corals. Previous approaches for modeling coral disease have not used powerful statistical inference methodologies to estimate parameters and for choosing the best model structure. Neither have they attempted to model the epidemic curve as it changes over a single season. In this study, however, a specially formulated maximum-likelihood fitting procedure, enabled us to estimate the most likely parameters in the model (*α* and *c*_*t*_), based on the disease dynamics in space and time. It also allowed us to link the spatiotemporal dynamics of the disease to seawater temperature (see *c*_*t*_ in Eqs 2 and 10) and gave us an opportunity to generate future projections that assess the impact of increasing SST on coral communities. Over any season, the spatial model revealed that as the temperature increases, the spread of WPD on corals looks similar to the spread of forest fires, where dense forests tend to burn completely while less dense forests are relatively resistant because the fire can hardly spread [80, 81].

Current assessments on the future of these reef-building corals are still relatively uncertain, being hindered by a lack of knowledge and understanding. In this context, our study exposes the critical importance of conducting additional multi-annual surveys on local spatial scales, for deepening our insights into these unique systems, and for supporting our efforts to successfully design effective conservation policies.

## Materials and Methods

### A Null Model for Testing the Spatiotemporal Pattern of WPD

Using a null model approach, which bases the null expectation on the spatial distribution of the entire pool of susceptible corals, we ascertained whether the *k* NICs found in the field were significantly aggregated around the PICs. We used *n*(*r*) (Eq 1) as a statistical index, defined as the mean number of NICs in a given month within a radius *r* from a PIC of the previous month. The non-aggregated null distribution of the NICs, and thus *n*(*r*), was generated as follows. Infected corals from the first month in each pair of sequential sampling dates defined the *m* fixed PICs. Then, via computer simulation, a group of *k* simulated NICs was randomly chosen from the entire pool of susceptible corals without any discrimination as to whether individuals were healthy or infected. *n*(*r*) was then determined for different radii *r*. This was repeated 1,000 times so that *n*(*r*) could be calculated for each group of *k* NICs for any value of *r*. These results made it possible to generate a 95% confidence interval (CI) envelope for *n*(*r*) under the null hypothesis of no aggregation of the NICs. We then calculated *n*(*r*) using only the *k* observed NICs found in the field. If the observed *n*(*r*) was found within the envelope, then the null hypothesis could not be rejected and the spatial distribution of NICs was considered independent of the spatial distribution of the PICs. Otherwise, if the observed *n*(*r*) was found outside the 95% CI envelope, the null hypothesis was rejected and the spatial distribution of NICs was considered significantly dependent on that of the PICs at *α* = 5% level (that is, the null hypothesis was rejected). NICs are considered spatially aggregated around PICs where the observed *n*(*r*) is greater than the null expectation, indicating inter-colonial (i.e. local) infections. On the other hand, NICs are considered over-dispersed in relation to PICs, if *n*(*r*) is smaller than the null expectation. This test was carried out for all pairs of sequential sampling dates.

### Testing the Validity of the Spatiotemporal SIS Model

To test whether the spatiotemporal model describes suitably the transmission pattern of the disease, we simulated the infection process at the studied site based on a given set of PICs for a particular date, using the most likely parameters $\left(\hat{\alpha },\;c_{1}(\hat{\alpha }),\ldots ,c_{11}(\hat{\alpha })\right)$. Thus, infected corals from the first month in each pair of sequential sampling dates define the *m* fixed PICs. Then, for a simulation that required a generation of new infections, we simply chose NICs at random from the entire pool of corals, assuming that coral-*i* has a probability *p*_*t*_(*i*) of being chosen (Eq 2). We repeated this process 1,000 times. Then, the model was tested for each pair of sequential sampling dates in two different ways: (i) the number of NICs observed in the field was compared with the distribution of the number of NICs obtained from the 1,000 random realizations; and (ii) the spatiotemporal index *n*(*r*) (Eq 1) that was calculated for the real data was compared with the distributions of *n*(*r*) that was calculated for any distance scale *r*, for the 1,000 random realizations. We tested whether the observed number of NICs and *n*(*r*) were significantly different from the null distribution of those simulated under a two-tailed test of 5% significance level. If this occurred it implied that the results found in the field are inconsistent with the proposed null model.

### The Epidemic Potential of WPD and *R*_*0*_

In the beginning of the transmission season, the spread of the disease in the local community exhibited epidemic-like growth. The epidemiological reproductive number, *R*_0_ [65], was calculated for the time period between June and August 2006 (the development period of the disease within the community), using the approximate relationship $R_{0}\approx e^{rT_{G}}$[82] (cf., Zvuloni *et al*. [16] for black-band disease (BBD)). The exponential growth rate is governed by the parameter *r*, which is estimated by fitting an exponential function to the (cumulative) incidence of the infective numbers. The parameter *T*_*G*_ is the observed mean generation interval, i.e., the interval between a coral becoming infected and its subsequent infection of another coral (see Zvuloni *et al*. [16]). *R*_0_ measures the epidemic potential of a pathogen and is defined as the mean number of secondary infections caused by a typical single infectious individual in a wholly susceptible coral community. When *R*_0_ ≤ 1, the introduction of an infected individual will fail to result in an outbreak. If, however, *R*_0_ > 1, then the introduction of the disease is likely to result in an epidemic that persists for extended periods.

### Simulating Future Projections of WPD

Linking the spatiotemporal model (Eq 2) to seawater temperatures allows us to assess the potential future impact of WPD on the local coral community. We calculated the probability of each susceptible coral to become infected according to Eq 2, where *c*_*t*_ in this equation was determined by fitting a quadratic model to fit SST according to the SST in that month (Eq 10). We set *α* = 1.9, which was found to be the best fitting exponent. The use of Eqs 2 and 10 for future predictions ensures that the probability of any susceptible coral to become infected has both spatial and seasonal/environmental components.

In accordance with our data, simulations are carried out in discrete time steps from month to month. For all simulated projections, we use the last month of the real data as initial conditions for the future projections, and SST is based on a time series measured between June 2006 and May 2007, which we assume repeats yearly. In light of global change, there is also obvious interest in trying to assess long-term effects of variations in SST, and we do this by varying the levels of SST in our simulated projections.

Each year in the beginning of the infection period we randomly infected one of the corals. This insured that the local population did not stay infection free due to stochastic fadeouts in the previous season. Clearance and death rates were month specific and calculated based on collected data, i.e., the probability of death, recovery, or remaining infected is determined by the fraction of infections that died, cleared, or stayed infected in the same month in the original data.

The locations for new recruits in the 10×10 m plot are randomly chosen anywhere on the plot whenever a recruitment event takes place. This approach sets no spatial restrictions on coral settlement, and as such does not constrain the topological distribution of the corals. We assume that the *per capita* recruitment is either: (i) “recruitment limited”—independent of local community density by assuming a constant influx of recruits per year. Alternatively, we assume recruitment is (ii) “free-space regulated”—dependent on the level of free substrate in the local patch; following from the hypothesis that this is a limiting resource in many marine benthic populations [66–69]. Here it is assumed that following a coral’s death, a healthy recruit instantaneously replaces it. In the first scenario (i), due to the spatial component of the model, the coral density may play a significant role in the transmission probability of the disease. On the other hand, in the second scenario (ii), the coral community density remains constant, and the role of the spatial component in the model is also expected to be relatively constant. In reality, coral recruitment is likely to lie somewhere between these two extremes, with variations in the location of different reefs along this continuum (for further reading on these assumptions, see [66–69].

## Supporting Information

S1 Fig Size structure of the studied coral community. **(a**) The coral community at the study site is extremely dense (>50 corals/m^2^). As reference, the distance between the two parallel lines is 1 m. (**b)** This community is composed of mostly relatively small massive corals, many of which are susceptible to infection by WPD (average of *ca*. 27.5 susceptible corals/m^2^). No differences were found between the size frequency distribution of susceptible vs. infected corals (*P*_*v*_ = 0.47; Kolmogorov-Smirnov two-sample test).
(PDF)

S2 Fig Plots of the spatiotemporal index *n*(*r*) calculated for pairs of sequential sampling dates (see text). The black line represents the observed *n*(*r*) values (Eq 1) for corals infected with white-plague disease (WPD). The shaded areas are the Monte Carlo 95% confidence interval (CI) envelopes, representing two different null expectations: **(a)** new infections develop randomly within the studied plot, independent of the spatial location of infected corals from the previous month; and **(b)** new infections develop according to the spatiotemporal model (Eq 2). For distance scales (*r*) where *n*(*r*) values fall within the envelope, the spatial distribution of infected corals does not differ significantly from the null distribution. Infected corals are significantly more aggregated (/over-dispersed) where the observed *n*(*r*) values fall above (/below) the CI envelope. In all cases the NICs observed in the field appeared to form aggregations around PICs over distance scales of up to 4.5 m. That is, in all cases the hypothesis that the NICs were infected by a random process of disease transmission independent of the spatial location of the PICs was rejected. Almost in all cases, the observed *n*(*r*) was purely within the expectation of the spatiotemporal model (Eq 2) for all distance scales *r*. However, in a few cases the observed *n*(*r*) was found to be greater than the upper bound of the 95% CI envelope generated by the model realizations for certain distance scales (see, for example, August-September 2006).
(PDF)

S3 Fig Probability surface plots for all pair of sequential sampling dates between June 2006 and May 2007. The probability of infection at each point within the 10×10 m studied site is displayed as a gradient of colors. Such that, warm colors (e.g. red) represent a high probability of infection (‘disease hotspots’) and cold colors (e.g. blue) represent a lower probability of infection. The probability was calculated by Eq 2 (using the best fitting parameters *α*, *c*_1_, *c*_2_,…, *c*_11_; see text) for a set of all Previously-Infected Corals (PICs; red circles) observed in the field. Note that in nearly all cases Newly-Infected Corals (NICs; white circles) develop in significant proximity to PICs as proposed by the model.
(PDF)

S4 Fig Simulated future projections of the local coral community. In **A)** are the number of infected corals, and in **B)** is the total community size of live corals. The simulated projections in red are equivalent to those in Fig 6 of the main text (where we used the exact values of estimated *c*_1_, *c*_2_,…, *c*_11_), and in green are an example where we allowed each of the parameters *c*_1_, *c*_2_,…, *c*_11_ to vary uniformly +/-2.5% from their original estimated values. We found the results to be equivalent demonstrating the robustness of our described patterns under mild parameter variation. To make this clearer, we show here a close up of the projections from year 25 to year 75. As in Fig 6, the simulations in panels **a**, **b** and **c** relied on the demographic scenario of constant influx of recruits (64 recruits per year), while in panels **d**, **e** and **f**, they rely on the scenario of free-space regulation of recruitment (see Material and Methods). Panels **a** and **d** are based on the SST time-series measured between June 2006 and May 2007 recurrently from year to year in the corresponding months. Based on this time-series, we generate future projections by adding 0.5°C (panels **b** and **e**) and 1°C (panels **c** and **f**) to the SST of each month. In these simulations we allow each new recruit to settle randomly anywhere on the 10×10 m plane.
(PDF)

S1 Table Maximum-likelihood estimates for the parameters *c*_*t*_ (*c*_1_, *c*_2_,…, *c*_11_) (see Eqs 3–9), constants that express the transmission strength of the disease during month *t*. (PDF)
