# Supplementary material for: Modeling the Impact of White-Plague Coral Disease in Climate Change Scenarios
Source: PLoS Comput Biol. 2015 Jun 18;11(6):e1004151. doi: 10.1371/journal.pcbi.1004151 (PMC4473065; doi:10.1371/journal.pcbi.1004151)
Supplement: S1 Fig — (a) The coral community at the study site is extremely dense (>50 corals/m2). As reference, the distance between the two parallel lines is 1 m. (b) This community is composed of mostly relatively small massive corals, many of which are susceptible to infection by WPD (average of ca. 27.5 susceptible corals/m2). No differences were found between the size frequency distribution of susceptible vs. infected corals (P v = 0.47; Kolmogorov-Smirnov two-sample test). (PDF) [file pcbi.1004151.s001.pdf]

Figure S1

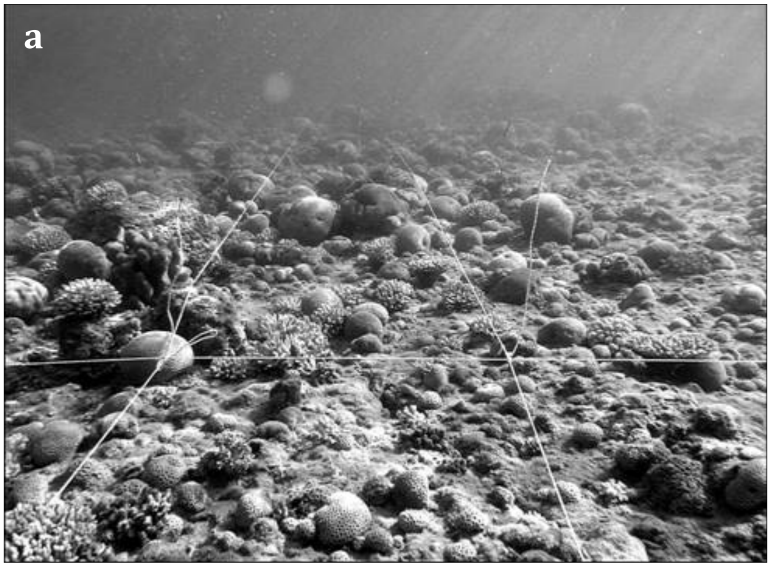

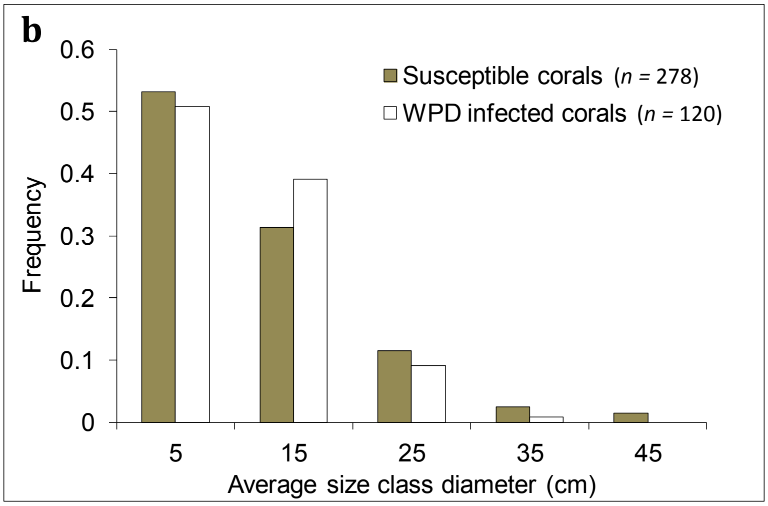

Figure S1. Size structure of the studied coral community. (a) The coral community at the study site is extremely dense (>50 corals/m^2). As reference, the distance between the two parallel lines is 1 m. (**b)** This community is composed of mostly relatively small massive corals, many of which are susceptible to infection by WPD (average of *ca.* 27.5 susceptible corals/m^2). No differences were found between the size frequency distribution of susceptible vs. infected corals (P_v = 0.47; Kolmogorov-Smirnov two-sample test).
