# Supplementary material for: Modeling the Impact of White-Plague Coral Disease in Climate Change Scenarios
Source: PLoS Comput Biol. 2015 Jun 18;11(6):e1004151. doi: 10.1371/journal.pcbi.1004151 (PMC4473065; doi:10.1371/journal.pcbi.1004151)
Supplement: S2 Fig — The black line represents the observed n(r) values (Eq 1) for corals infected with white-plague disease (WPD). The shaded areas are the Monte Carlo 95% confidence interval (CI) envelopes, representing two different null expectations: (a) new infections develop randomly within the studied plot, independent of the spatial location of infected corals from the previous month; and (b) new infections develop according to the spatiotemporal model (Eq 2). For distance scales (r) where n(r) values fall within the envelope, the spatial distribution of infected corals does not differ significantly from the null distribution. Infected corals are significantly more aggregated (/over-dispersed) where the observed n(r) values fall above (/below) the CI envelope. In all cases the NICs observed in the field appeared to form aggregations around PICs over distance scales of up to 4.5 m. That is, in all cases the hypothesis that the NICs were infected by a random process of disease transmission independent of the spatial location of the PICs was rejected. Almost in all cases, the observed n(r) was purely within the expectation of the spatiotemporal model (Eq 2) for all distance scales r. However, in a few cases the observed n(r) was found to be greater than the upper bound of the 95% CI envelope generated by the model realizations for certain distance scales (see, for example, August-September 2006). (PDF) [file pcbi.1004151.s002.pdf]

Figure S2

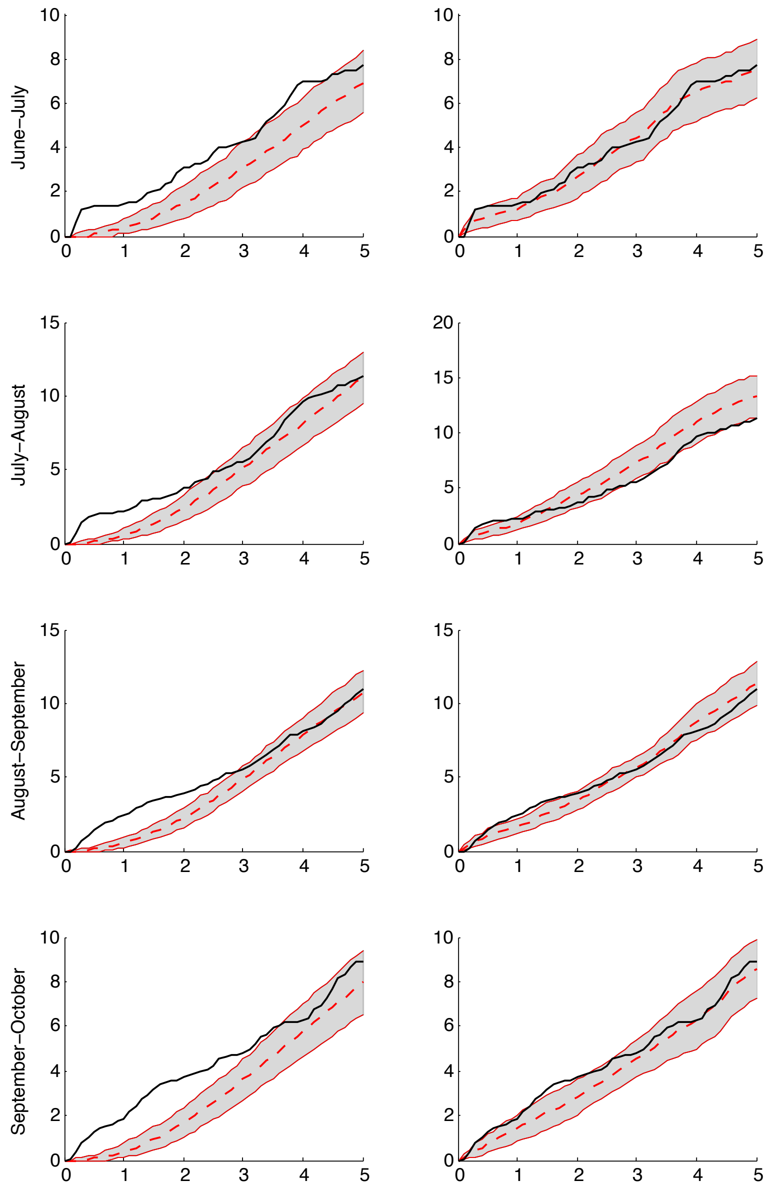

Figure S2 continued

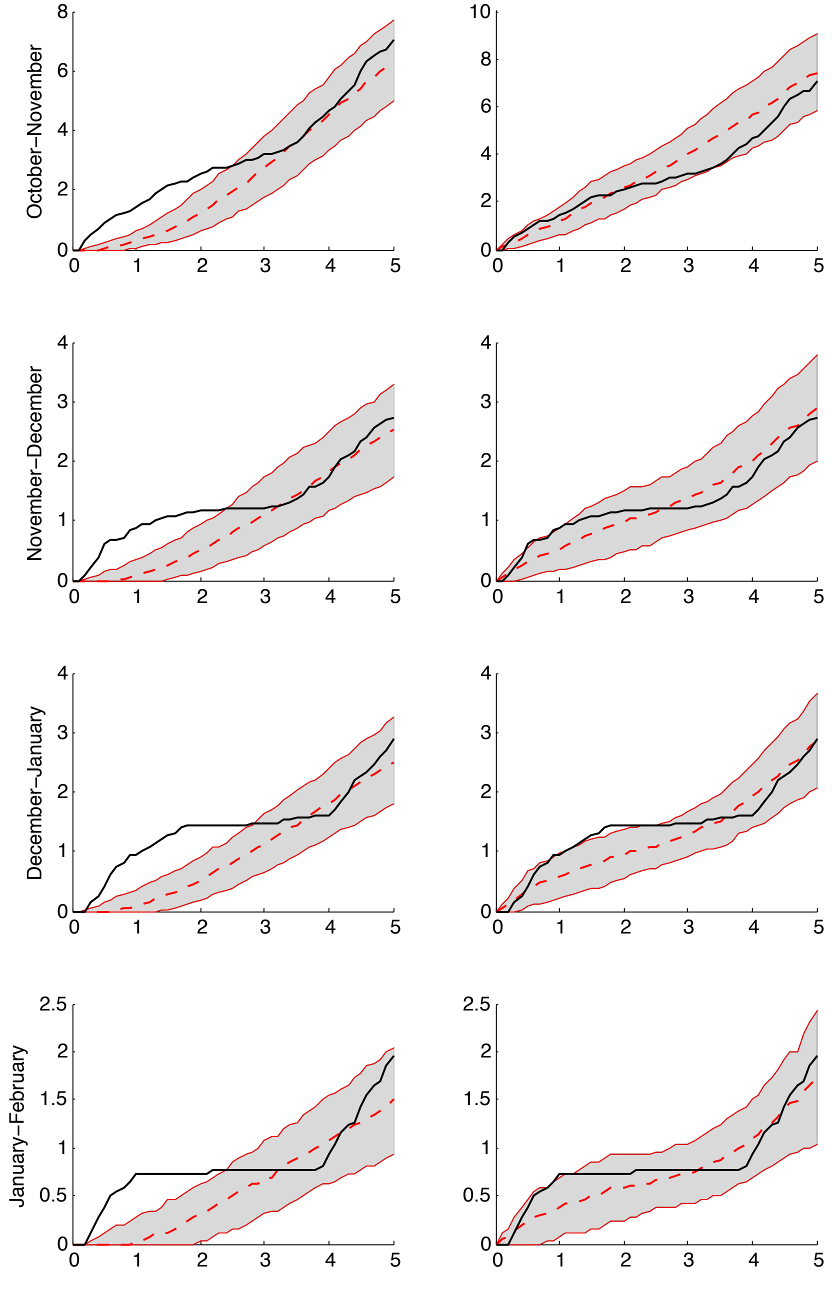

Figure S2 continued

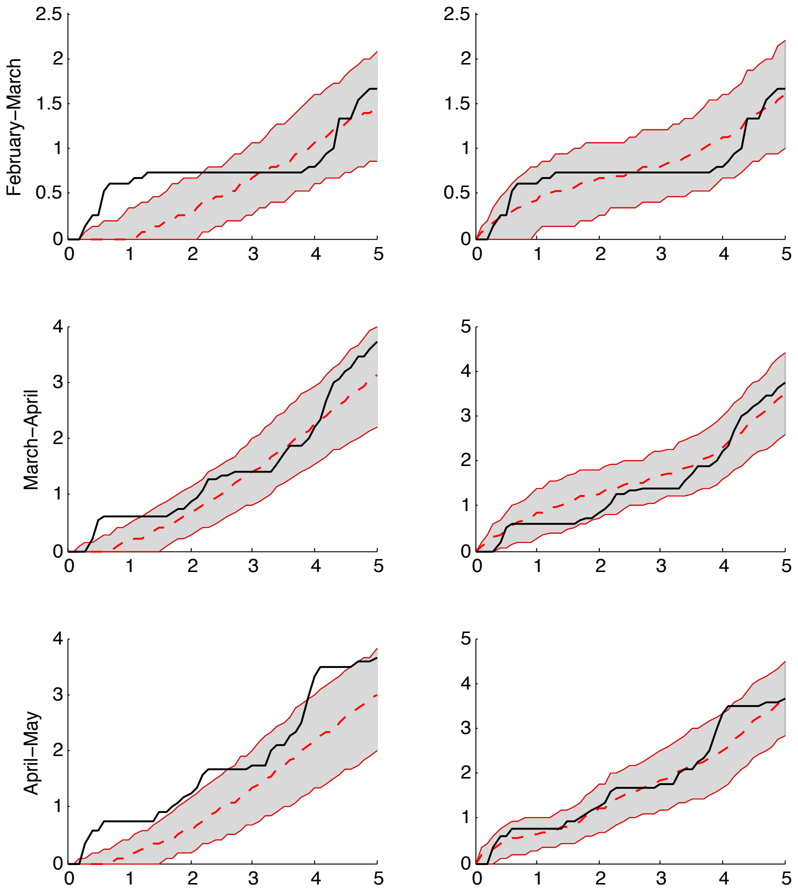

Figure S2. Plots of the spatiotemporal index *n*(*r*) calculated for pairs of sequential sampling dates (see text). The black line represents the observed *n*(*r*) values (eqn 1) for corals infected with white-plague disease (WPD). The shaded areas are the Monte Carlo 95% confidence interval (CI) envelopes, representing two different null expectations: **(a)** new infections develop randomly within the studied plot, independent of the spatial location of infected corals from the previous month; and **(b)** new infections develop according to the spatiotemporal model (eqn 2). For distance scales (*r*) where *n*(*r*) values fall within the envelope, the spatial distribution of infected corals does not differ significantly from the null distribution. Infected corals are significantly more aggregated (/over-dispersed) where the observed *n*(*r*) values fall above (/below) the CI envelope.

In all cases the NICs observed in the field appeared to form aggregations around PICs over distance scales of up to 4.5 m. That is, in all cases the hypothesis that the NICs were infected by a random process of disease transmission independent of the

spatial location of the PICs was rejected. Almost in all cases, the observed $n(r)$ was purely within the expectation of the spatiotemporal model (eqn 2) for all distance scales r. However, in a few cases the observed $n(r)$ was found to be greater than the upper bound of the 95% CI envelope generated by the model realizations for certain distance scales (see, for example, August-September 2006).
