# Supplementary material for: Modeling the Impact of White-Plague Coral Disease in Climate Change Scenarios
Source: PLoS Comput Biol. 2015 Jun 18;11(6):e1004151. doi: 10.1371/journal.pcbi.1004151 (PMC4473065; doi:10.1371/journal.pcbi.1004151)
Supplement: S3 Fig — The probability of infection at each point within the 10×10 m studied site is displayed as a gradient of colors. Such that, warm colors (e.g. red) represent a high probability of infection (‘disease hotspots’) and cold colors (e.g. blue) represent a lower probability of infection. The probability was calculated by Eq 2 (using the best fitting parameters α, c 1, c 2,…, c 11; see text) for a set of all Previously-Infected Corals (PICs; red circles) observed in the field. Note that in nearly all cases Newly-Infected Corals (NICs; white circles) develop in significant proximity to PICs as proposed by the model. (PDF) [file pcbi.1004151.s003.pdf]

Figure S3

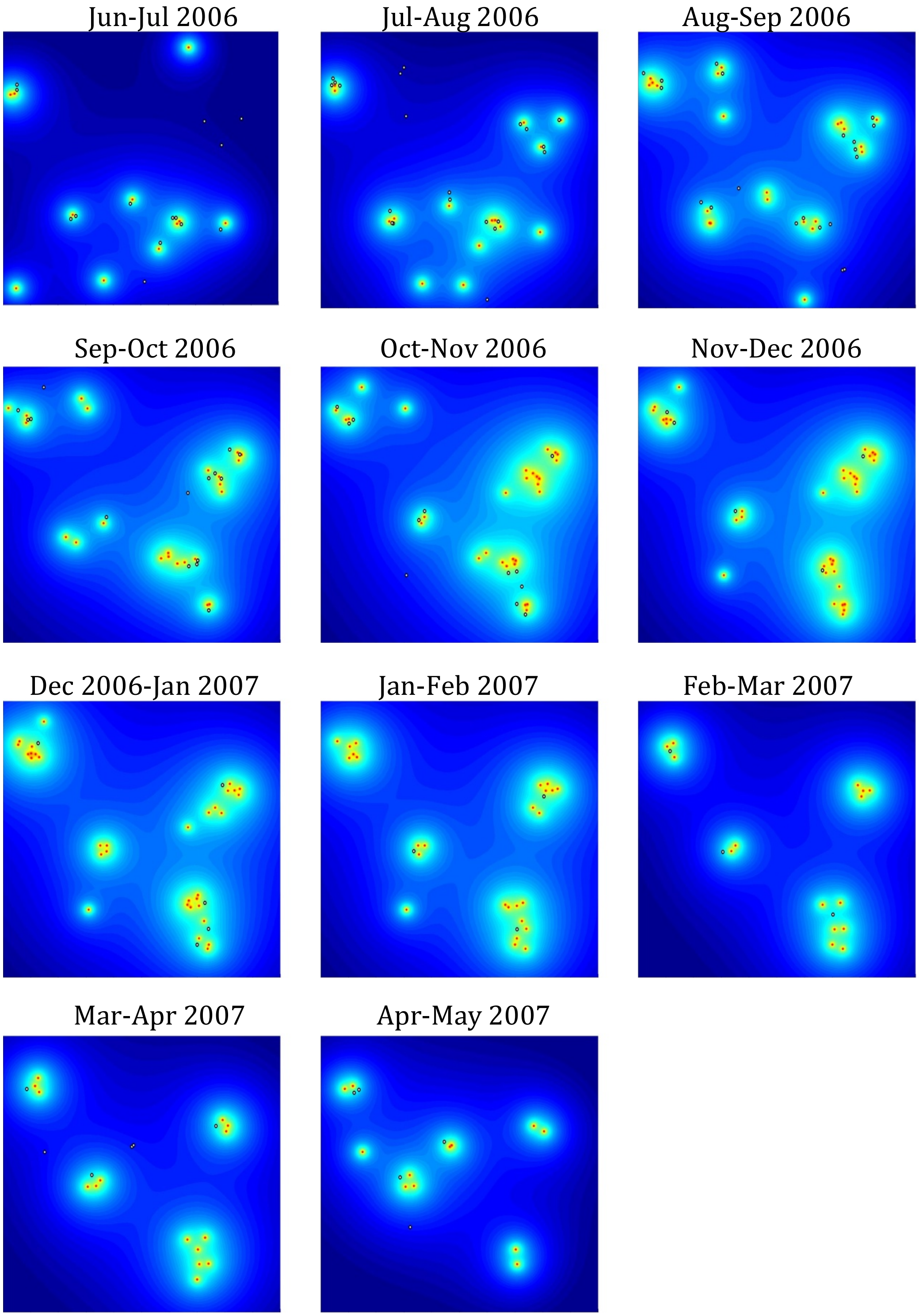

Jun-Jul 2006
Jul-Aug 2006
Aug-Sep 2006
Sep-Oct 2006
Oct-Nov 2006
Nov-Dec 2006
Dec 2006-Jan 2007
Jan-Feb 2007
Feb-Mar 2007
Mar-Apr 2007
Apr-May 2007

Figure S3. Probability surface plots for all pair of sequential sampling dates between June 2006 and May 2007. The probability of infection at each point within the 10×10 m studied site is displayed as a gradient of colors. Such that, warm colors (e.g. red) represent a high probability of infection ('disease hotspots') and cold colors (e.g. blue) represent a lower probability of infection. The probability was calculated by eqn 2 (using the best fitting parameters $\alpha, c_1, \cdots, c_{11}$; see text) for a set of all Previously-Infected Corals (PICs; red circles) observed in the field. Note that in nearly all cases Newly-Infected Corals (NICs; white circles) develop in significant proximity to PICs as proposed by the model.
