# Supplementary material for: Modeling the Impact of White-Plague Coral Disease in Climate Change Scenarios
Source: PLoS Comput Biol. 2015 Jun 18;11(6):e1004151. doi: 10.1371/journal.pcbi.1004151 (PMC4473065; doi:10.1371/journal.pcbi.1004151)
Supplement: S4 Fig — In A) are the number of infected corals, and in B) is the total community size of live corals. The simulated projections in red are equivalent to those in Fig 6 of the main text (where we used the exact values of estimated c 1, c 2,…, c 11), and in green are an example where we allowed each of the parameters c 1, c 2,…, c 11 to vary uniformly +/-2.5% from their original estimated values. We found the results to be equivalent demonstrating the robustness of our described patterns under mild parameter variation. To make this clearer, we show here a close up of the projections from year 25 to year 75. As in Fig 6, the simulations in panels a, b and c relied on the demographic scenario of constant influx of recruits (64 recruits per year), while in panels d, e and f, they rely on the scenario of free-space regulation of recruitment (see Material and Methods). Panels a and d are based on the SST time-series measured between June 2006 and May 2007 recurrently from year to year in the corresponding months. Based on this time-series, we generate future projections by adding 0.5°C (panels b and e) and 1°C (panels c and f) to the SST of each month. In these simulations we allow each new recruit to settle randomly anywhere on the 10×10 m plane. (PDF) [file pcbi.1004151.s004.pdf]

Figure S4

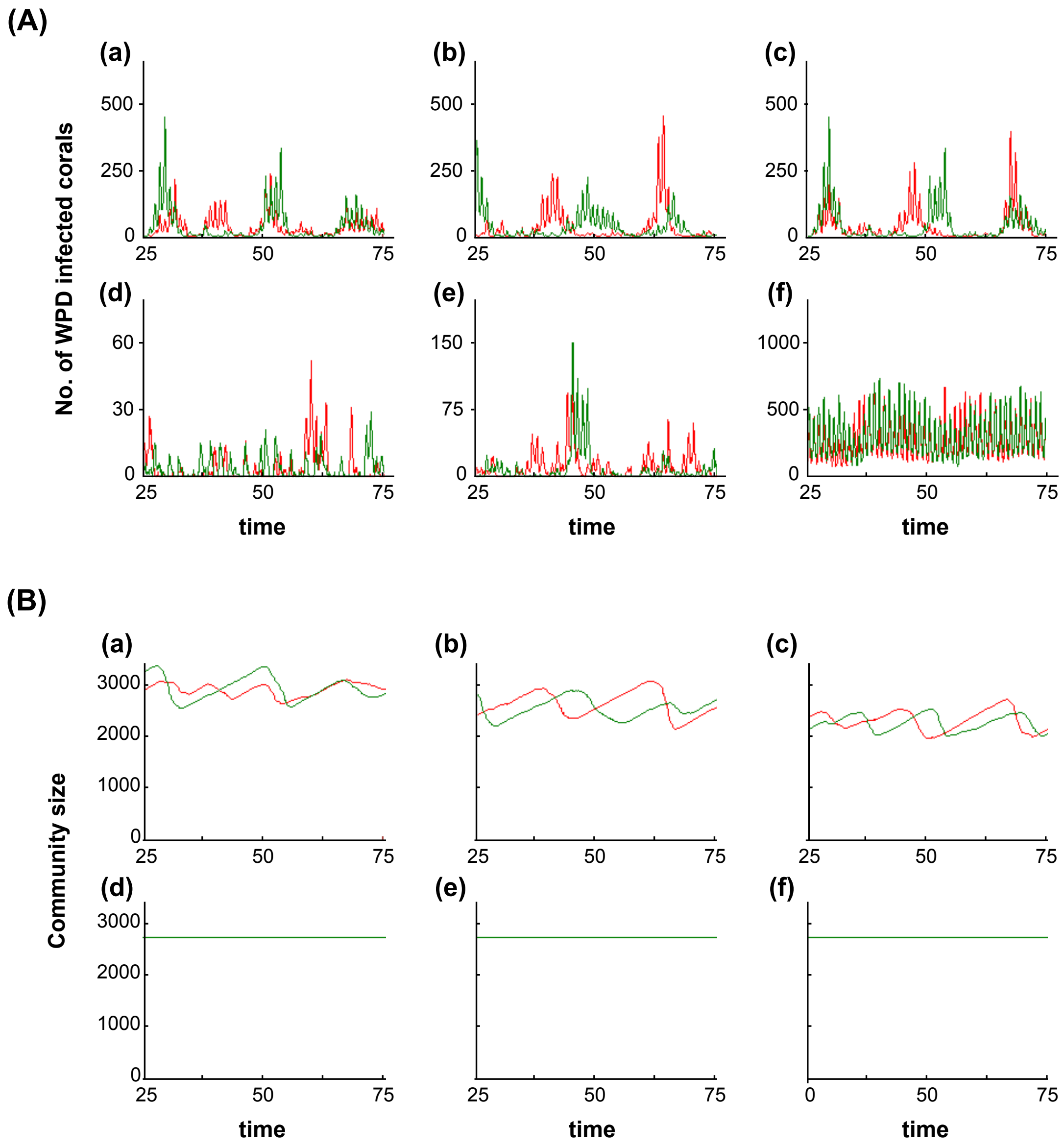

Figure S4. Simulated future projections of the local coral community: In **A)** are the number of infected corals, and in **B)** is the total community size of live corals. The simulated projections in red are equivalent to those in Figure 6 of the main text (where we used the exact values of estimated c(1),c(2)...,c(11)), and in green are an example where we allowed each of the parameters c(1),c(2)...,c(11) to vary uniformly +/-2.5% from their original estimated values. We found the results to be

equivalent demonstrating the robustness of our described patterns under mild parameter variation. To make this clearer, we show here a close up of the projections from year 25 to year 75. As in Figure 6, the simulations in panels **a**, **b** and **c** relied on the demographic scenario of constant influx of recruits (64 recruits per year), while in panels **d**, **e** and **f**, they rely on the scenario of free-space regulation of recruitment (see *Materials and Methods*). Panels **a** and **d** are based on the SST time-series measured between June 2006 and May 2007 recurrently from year to year in the corresponding months. Based on this time-series, we generate future projections by adding 0.5^{o}C (panels **b** and **e**) and 1°C (panels **c** and **f**) to the SST of each month. In these simulations we allow each new recruit to settle randomly anywhere on the 10×10 m plane.
