# Supplementary material for: Modeling the Impact of White-Plague Coral Disease in Climate Change Scenarios
Source: PLoS Comput Biol. 2015 Jun 18;11(6):e1004151. doi: 10.1371/journal.pcbi.1004151 (PMC4473065; doi:10.1371/journal.pcbi.1004151)
Supplement: S1 Table — (PDF) [file pcbi.1004151.s005.pdf]

Table S1. Maximum-likelihood estimates for the parameters c_t ($c_1, c_2, \cdots, c_{11}$) (see equations 3-9), constants that express the transmission strength of the disease during month *t*.

Jul 2006	Aug 2006	Sep 2006	Oct 2006	Nov 2006	Dec 2006	Jan 2007	Feb 2007	Mar 2007	Apr 2007	May 2007
c_1	c_2	c_3	c_4	c_5	c_6	c_7	c_8	c_9	c_{10}	c_{11}
0.0014	0.0016	0.0014	0.0007	0.0007	0.0002	0.0002	0.0001	0.0002	0.0005	0.0004
